# Development of an alcoholic liver disease model for drug evaluation from human induced pluripotent stem cell-derived liver organoids: ALD model from hiPSC-derived liver organoids

**DOI:** 10.3724/abbs.2024074

**Published:** 2024-05-30

**Authors:** Zhiwei Feng, Bingrui Zhou, Qizhi Shuai, Yunliang Wei, Ning Jin, Xiaoling Wang, Hong Zhao, Zhizhen Liu, Jun Xu, Jianbing Mu, Jun Xie

**Affiliations:** 1 Department of Biochemistry and Molecular Biology Shanxi Key Laboratory of Birth Defect and Cell Regeneration MOE Key Laboratory of Coal Environmental Pathogenicity and Prevention Shanxi Medical University Taiyuan 030001 China; 2 Department of Hepatobiliary and Pancreatic Surgery and Liver Transplant Center the First Hospital of Shanxi Medical University Taiyuan 030001 China; 3 Laboratory of Malaria and Vector Research National Institute of Allergy and Infectious Diseases National Institutes of Health 12735 Twinbrook Parkway USA

**Keywords:** human induced pluripotent stem cell, liver organoid, alcoholic liver disease, drug screening

## Abstract

Alcoholic liver disease (ALD) poses a significant health challenge, so comprehensive research efforts to improve our understanding and treatment strategies are needed. However, the development of effective treatments is hindered by the limitation of existing liver disease models. Liver organoids, characterized by their cellular complexity and three-dimensional (3D) tissue structure closely resembling the human liver, hold promise as ideal models for liver disease research. In this study, we use a meticulously designed protocol involving the differentiation of human induced pluripotent stem cells (hiPSCs) into liver organoids. This process incorporates a precise combination of cytokines and small molecule compounds within a 3D culture system to guide the differentiation process. Subsequently, these differentiated liver organoids are subject to ethanol treatment to induce ALD, thus establishing a disease model. A rigorous assessment through a series of experiments reveals that this model partially recapitulates key pathological features observed in clinical ALD, including cellular mitochondrial damage, elevated cellular reactive oxygen species (ROS) levels, fatty liver, and hepatocyte necrosis. In addition, this model offers potential use in screening drugs for ALD treatment. Overall, the liver organoid model of ALD, which is derived from hiPSC differentiation, has emerged as an invaluable platform for advancing our understanding and management of ALD in clinical settings.

## Introduction

A report from the World Health Organization indicated that alcohol abuse led to more than 3 million deaths in 2018, accounting for 1 in 20 deaths and contributing to more than 5% of the global disease burden. Prolonged alcohol abuse can lead to the development of alcoholic liver disease (ALD), which encompasses fatty liver disease (steatosis), alcoholic hepatitis, and alcoholic cirrhosis [ 1, 2]. The pathological features of ALD include hepatocyte steatosis, oxidative stress, inflammation, and liver fibrosis. Disease progression can lead to hepatocyte necrosis and apoptosis, liver cirrhosis, and even liver cancer [ 3, 4]. At present, the models used to study ALD mainly include animal models and liver cell line culture models [ 5, 6]. Two-dimensional cultures of liver cell lines have traditionally been used to mimic diseases and develop medications. For instance, flat cultures of liver stellate cells (HSCs) have been employed, with or without the addition of soft hydrogels such as polyacrylamide gels. However, the genetic profiles of these cells often do not align with those of cirrhotic tissues in humans. Translating findings from 2D cultures has been challenging due to their inability to replicate natural characteristics such as dynamic physical and chemical signals and the microenvironmental structures present in the liver lobule, which often leads to a rapid decline in liver cell function [7]. Therefore, the application of these models in ALD research is limited due to species differences and the constraints of two-dimensional (2D) culture [ 8– 10].

Organoids refer to the self-assembly of adult stem cells or pluripotent stem cells in a 3D *in vitro* culture environment to form tissue analogues with 3D structures that accurately reflect the characteristics of the original tissue (derived from adult stem cells) or directed differentiated tissue (derived from induced pluripotent stem cells) [11]. Organoids originated from iPSCs are widely recognized as pivotal elements in the field of disease modelling based on organoids. Recent studies have demonstrated the successful development of vascularized organoids derived from iPSCs. In a specific study, the integration of stromal elements such as vasculature, fibroblasts, and immune cells was achieved by utilizing mesoderm progenitor cells induced from iPSCs [12]. Organoid culture offers the potential for long-term *in vitro* culture expansion while maintaining stable genetic characteristics, thus providing a solution to the challenges of liver disease models [13]. *In vitro* organoid models represent a significant advancement for translational research, as these 3D models closely mimic *in vivo* biological processes such as tissue renewal and the response of tissues to drugs, toxins, and mutagenesis. Compared to traditional monolayer cultures, 3D liver organoid cultures offer more accurate models for studying hepatic toxicity.

In the present study, we initially differentiated hiPSCs into liver organoids. The structural integrity, cellular composition, expression of liver-related proteins, and specific liver functions of these derived liver organoids were assessed. Subsequently, the liver organoids were subject to treatment with different concentrations of ethanol to establish an ALD model. This model successfully recapitulates key pathological features of ALD, including hepatocyte adiposeness, mitochondrial damage, elevated cellular ROS, and cell death. Finally, we selected three drugs with reported therapeutic and preventive potential for ALD, utilizing the established liver organoid model as a platform for drug screening and evaluating its applicability for clinical ALD studies.

## Materials and Methods

### Cell lines and cell culture

The human induced pluripotent stem cell line hiPSC-B1 was purchased from Cellapy ^®^ (CA4025106; Beijing, China) and cultured on plates coated with low growth factor Matrigel (354230; Corning, New York, USA) using mTeSR ^TM^ 1 medium (85850; StemCell, Vancouver, Canada) supplemented with 100 U/mL penicillin/streptomycin (Solarbio, Beijing, China). The culture was maintained in a humidified atmosphere containing 5% CO _2_ at 37°C in a cell incubator (Eppendorf, Mittelsachsen, Germany). The medium was changed daily, and the cells were passaged at a ratio of 1:5‒1:8 every 4‒7 days.

### Liver organoid generation

Single undifferentiated hiPSC-B1 cells at passages 30–50 were digested with Accutase (00-4555; Thermo Fisher Scientific, Waltham, USA) and seeded onto Matrigel-coated culture plates. Once the cells reached approximately 80% confluence without signs of differentiation, the differentiation process was initiated. The medium was replaced by RPMI 1640 medium (31800; Solarbio) containing 100 ng/mL activin A (120-14E; PeproTech, Rocky Hill, USA) and 50 ng/mL bone morphogenetic protein 4 (BMP4; 314-BP, R&D Systems, Minneapolis, USA) on day 1, followed by RPMI 1640 medium containing 100 ng/mL activin A and 0.2% knockout serum replacement (KSR; A3181502; Gibco, Carlsbad, USA) on day 2, and RPMI 1640 medium containing 100 ng/mL activin A and 2% KSR on day 3. From day 4 to day 6, the cells were cultured in high-glucose DMEM (SC102-02; Sevenbio, Beijing, China) supplemented with 50 ng/mL recombinant human fibroblast growth factor 10 (FGF-10; 100-26; PeproTech), 3 μM CHR99021 (SML1046; Sigma, St Louis, USA), 10% KSR, and 1% NEAA (11140; Gibco). The cells were maintained at 37°C in 5% CO _2_ with 95% air in the cell incubator, and the medium was changed daily. By approximately day 6, spheroids of the foregut endoderm formed on the plate. On day 7, the spheroids were dissociated with Accutase into single cells, embedded in 100% Matrigel drops on plates in high-glucose DMEM supplemented with 3 μM CHR99021, 5 ng/mL FGF-2 (100-18B; PeproTech), 0.5 μM A83-01 (HY10432; MCE, Monmouth Junction, USA), 20 ng/mL recombinant human epidermal growth factor (EGF; AF100-15; PeproTech), 10% KSR and 1% NEAA, and cultured for 2 days. On day 9, the medium was replaced by high-glucose DMEM containing 2 μM retinoic acid (RA; R2625; Sigma), 10% KSR, and 1% NEAA, and the cells were cultured for 4 days, with medium renewal on day 11. After RA treatment, liver organoids gradually formed. On day 13, the medium was replaced by hepatocyte culture medium (HCM; CC-3198; Lonza, Visp, Switzerland) supplemented with 10 ng/mL hepatocyte growth factor (HGF; 100-39; PeproTech), 0.1 mM dexamethasone (Dex; D4902; Sigma) and 20 ng/mL oncostatin M (OSM; 300-10; PeproTech). Liver organoids were isolated from Matrigel by scraping and pipetting and passaged on day 16, and the medium was changed every 3 days. The liver organoids were ready for subsequent applications after days 22‒25.

### RNA isolation, reverse transcription PCR, and quantitative PCR

Total RNA was extracted from liver organoids using RNAiso Plus reagent (TaKaRa, Tokyo, Japan) in combination with chloroform and isopropanol. Complementary DNA (cDNA) was synthesized using the PrimeScript™ RT Reagent kit with gDNA Eraser (TaKaRa) following the manufacturer’s protocol. qPCR was performed on a QuantStudio 3 Real-Time PCR System (Applied Biosystems, Foster City, USA) using SYBR ^®^ Premix Ex Taq ^TM^ II (TaKaRa). All primer information for each target gene was obtained from the PrimerBank website ( https://pga.mgh.harvard.edu/primerbank/). The PCR conditions were as follows: initial denaturation at 95°C for 1 min, followed by 40 cycles of 95°C for 5 s and 60°C for 30 s, with a final melting curve stage. All samples were run in triplicate concurrently. The values were normalized to those of the internal control products of glyceraldehyde-3-phosphate dehydrogenase ( *GAPDH*). A complete list of the primers used is shown in Supplementary Table S1.

### Immunofluorescence (IF) staining

Immunofluorescence staining was conducted according to a previously described protocol [14]. Liver organoids were fixed in 4% PFA for 4 h, dehydrated, embedded in paraffin, and sectioned at 4-μm thickness. The paraffin-embedded sections were then dewaxed with xylene, rehydrated, and subject to heat-mediated antigen retrieval by microwaving for 15 min in Tris-EGTA buffer (pH 9.0). Following blocking with 1% BSA/PBS (Sigma) for 1 h, the sections were incubated with primary antibodies in 1% BSA/PBS at 4°C overnight and with secondary antibodies in 1% BSA/PBS for 1 h in the dark at room temperature (RT). A complete list of primary and secondary antibodies used is provided in Supplementary Table S2. Subsequently, 4′,6-diamidino-2-phenylindole (DAPI; Abcam, Cambridge, UK) was used to counterstain the nuclei. After they were incubated with primary and secondary antibodies and subject to DAPI staining, the sections were washed 3 times with PBS (5 min each time). Immunofluorescence images were acquired using a fluorescence microscope (Nikon, Tokyo, Japan). NIS-Elements Viewer software (Nikon) was used for image processing.

### Flow cytometry

Liver organoids were treated with trypsin EDTA (0.25%; Solarbio) for 30 min and dissociated into single cells, which were then passed through 70-μm cell strainers. Subsequently, the HLO cells were fixed in 4% PFA for 20 min, permeabilized in 0.2% Triton X/PBS for 10 min, blocked with 1% BSA/PBS blocking solution for 30 min, and then incubated with primary antibodies for 1 h. Following incubation with secondary antibodies for 0.5 h in the dark, the stained cells were analyzed using BD FACS Celesta flow cytometer (BD Biosciences, Franklin Lakes, USA), and the obtained data were analyzed using FlowJo 10.0 software (Stanford University, Stanford, USA). A complete list of primary and secondary antibodies used is provided in Supplementary Table S2.

### Western blot analysis

Total proteins were extracted from the liver organoids using cell lysis buffer (Solarbio) supplemented with protease inhibitors at 4°C, and the concentration of the protein samples was quantified using a BCA protein assay kit (EpiZyme, Shanghai, China). The protein samples were separated by 10% sodium dodecyl sulfate-polyacrylamide gel electrophoresis (SDS-PAGE) and transferred onto polyvinylidene fluoride (PVDF) membranes (Millipore, Billerica, USA). After being blocked with 5% non-fat milk for 1 h at room temperature, the PVDF membranes were incubated with diluted primary antibodies at 4°C overnight. Then, the membranes were washed 3 times with phosphate-buffered saline supplemented with Tween-20 (PBST) and incubated with peroxidase-conjugated goat anti-rabbit or mouse secondary antibodies (Abmart, Shanghai, China) at room temperature for 2 h. After three times wash with PBST, the proteins on the PVDF membrane were detected using an enhanced chemiluminescence (ECL) solution (EpiZyme, Shanghai, China) in a ChemiDoc MP electrophoretic imaging system (Bio-Rad, Hercules, USA), and the protein levels were quantified using Fiji software. A complete list of primary and secondary antibodies used is provided in Supplementary Table S2.

### Indocyanine green (ICG) uptake and release assay

The liver organoids were incubated with 1 mg/mL indocyanine green (ICG; Yuanye Bio, Shanghai, China) in medium for 30 min at 37°C. Following incubation, the medium containing ICG was discarded, and the organoids were washed 3 times with PBS. The uptake of ICG was examined under an inverted microscope (Leica, Wetzlar, Germany). Subsequently, the organoids were returned to the culture medium and incubated for an additional 48 h to assess the release of cellular ICG.

### Periodic acid-Schiff staining, Sirius red staining, Masson’s staining

Paraffin-embedded sections of liver organoid were subject to staining with periodic acid-Schiff (PAS; Servicebio, Wuhan, China), Sirius red (G-clone, Beijing, China), and Masson’s trichrome (Servicebio) according to the manufacturer’s instructions. The collagen volume fraction (CVF) was quantified using Fiji software.

### Intracellular lipid assays

The intracellular neutral lipid contents were measured using the lipophilic fluorescent probe dipyrromethene boron difluoride (BODIPY; GLPBIO, Tianjin, China) and the nuclear dye Hoechst 33342 (Solarbio). Liver organoids were dissociated into single organoids and incubated in hepatocyte culture medium (HCM) supplemented with 2 μM BODIPY and 10 μM Hoechst 33342 for 20 min at 37°C in the dark. After incubation, the organoids were washed three times with PBS, resuspended in PBS supplemented with Antifade Mounting Medium (Absin, Shanghai, China), and observed using inverted fluorescence microscope. BODIPY was visualized using the green fluorescence channel, while Hoechst 33342 was visualized using the blue fluorescence channel. The organoid suspension was then transferred to a 96-well or 384-well microtiter plate, and the fluorescence intensity was detected using a SpectraMax iD3 microplate reader (Molecular Devices, San Jose, USA). The excitation wavelength and emission wavelength of Hoechst 33342 were 350 nm and 460 nm, respectively. The excitation and emission wavelengths of BODIPY were 488 nm and 530 nm, respectively. Intracellular neutral lipid accumulation is indicated by an increase in the green/blue fluorescence intensity ratio.

### Mitochondrial membrane potential (MMP) assay

Alterations in the mitochondrial membrane potential were detected using a JC-1 kit (Solarbio) according to the manufacturer’s instructions. Liver organoids were dissociated into single organoids and incubated with JC-1 working solution for 30 min at 37°C in the dark. Afterward, the cells were washed three times with JC-1 buffer, resuspended in PBS supplemented with Antifade Mounting Medium, and observed using an inverted fluorescence microscope. The JC-1 monomer was visualized with the green fluorescence channel, while JC-1 aggregates were observed using the red fluorescence channel. The organoid suspension was then transferred to a 96-well or 384-well microtiter plate, and the fluorescence intensity was detected on a SpectraMax iD3 microplate reader. The excitation wavelength and emission wavelength of the JC-1 monomer were 488 nm and 530 nm, respectively. The excitation and emission wavelengths of the JC-1 aggregates were 529 nm and 590 nm, respectively. Mitochondrial damage is indicated by a decrease in the red/green fluorescence intensity ratio.

### Intracellular reactive oxygen species (ROS) assays

The levels of intracellular ROS were measured using the ROS probe dichlorodihydrofluorescein diacetate (DCFH-DA; Beyotime Biotech, Shanghai, China) and the nuclear dye Hoechst 33342. Liver organoids were dissociated into single organoids and incubated with HCM containing 10 μM DCFH-DA and 10 μM Hoechst 33342 for 30 min at 37°C in the dark. After incubation, the organoids were washed three times and resuspended in PBS supplemented with Antifade Mounting Medium before being observed under an inverted fluorescence microscope. DCFH-DA was visualized with the green fluorescence channel, and Hoechst 33342 was visualized with the blue fluorescence channel. The organoid suspension was then transferred to a 96-well or 384-well microtiter plate, and the fluorescence intensity was detected on a SpectraMax iD3 microplate reader. The excitation wavelength and emission wavelength of Hoechst 33342 were 350 nm and 460 nm, respectively. The excitation and emission wavelengths of DCFH-DA were 488 nm and 525 nm, respectively. The ROS level is indicated by changes in the green/blue fluorescence intensity ratio.

### Cell viability assays

The viability of liver organoids was assessed using a Calcein/PI Cell Viability/Cytotoxicity Assay kit (Beyotime Biotech) according to the manufacturer’s instructions. Liver organoids were dissociated into single organoids and incubated with Calcein AM/PI working solution for 30 min at 37°C in the dark. Following incubation, Antifade Mounting Medium was added, and the organoids were observed by inverted fluorescence microscopy. Calcein AM was visualized using the green fluorescence channel, while PI was visualized using the red fluorescence channel. The organoid suspension was then transferred to a 96-well or 384-well microtiter plate, and the fluorescence intensity was detected on a SpectraMax iD3 microplate reader. The excitation wavelength and emission wavelength of Calcein AM were 494 nm and 530 nm, respectively, while the excitation and emission wavelengths of PI were 535 nm and 617 nm, respectively. Changes in the green/red fluorescence intensity ratio indicate the cell viability of liver organoids.

### Statistical analysis

Data were analyzed using GraphPad Prism 8.0 statistical software (GraphPad, San Diego, USA) and are expressed as the mean±standard error of the mean (SEM). Student’s *t* test was used to evaluate the differences between two groups. One-way analysis of variance (ANOVA) was applied to compare the differences among three or more groups, with the Dunnett test used for comparisons between multiple experimental and control groups. A *P* value less than 0.05 was considered statistically significant.

## Results

### Generation of liver organoids from hiPSCs

**Figure 1:**
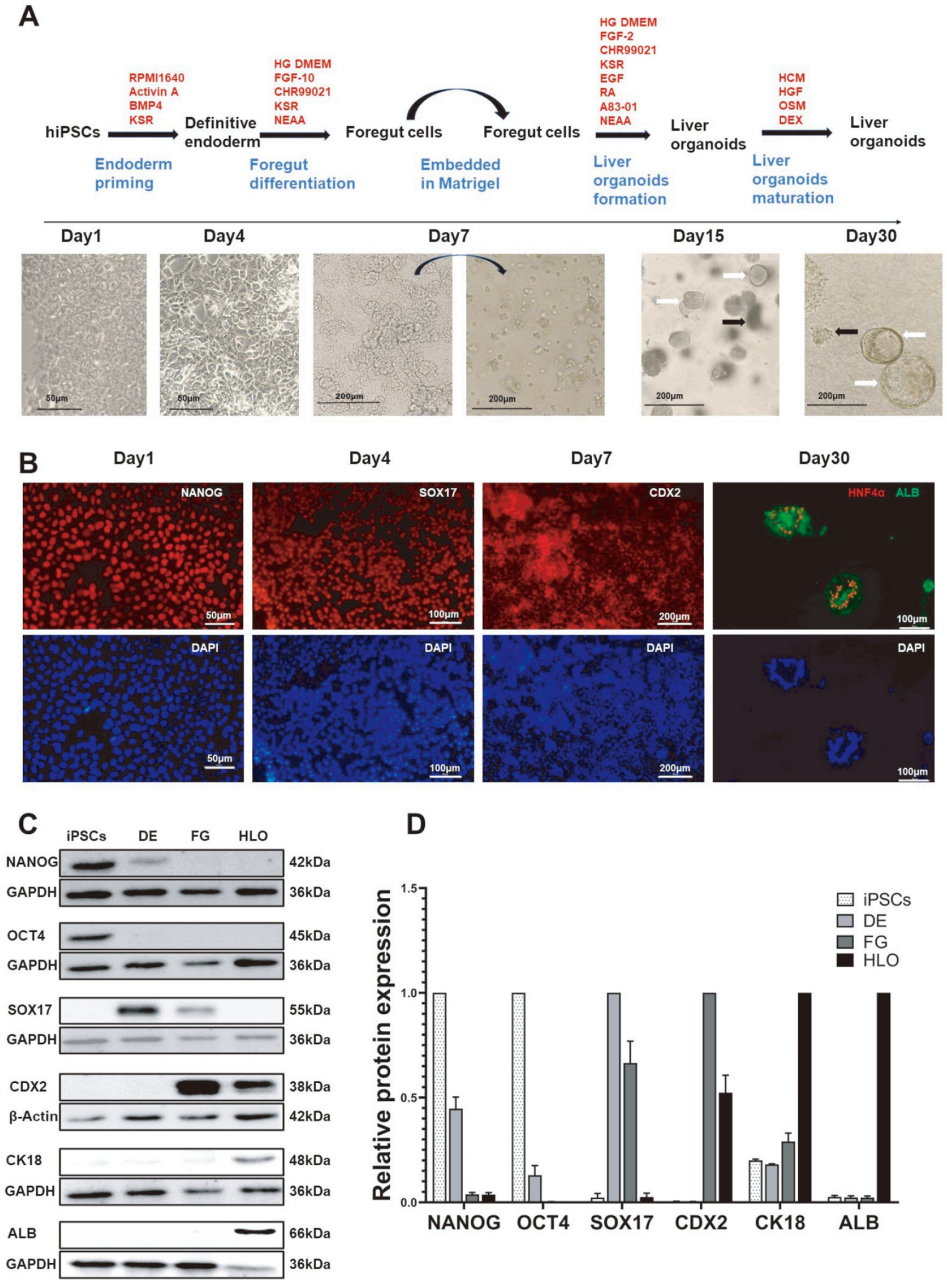
Figure 1 Differentiation of hiPSCs into liver organoids (A) Schematic diagram of the protocol used to induce the differentiation of liver organoids and morphological diagrams of the different stages of differentiation. The white arrows show a spherical mass, and the black arrows show an irregular mass in the bright field image on days 15 and 30. (B) Immunofluorescence staining images showing the expressions of the pluripotent stem cell marker protein NANOG, the definitive endoderm cell-specific protein SOX17, the foregut cell-specific protein CDX2, and the hepatocyte-specific proteins HNF4α and ALB at different stages of liver organoid differentiation. (C) Western blot analysis revealed that the pluripotent stem cell marker proteins OCT4 and NANOG, the definitive endoderm cell-specific protein SOX17, the foregut cell-specific protein CDX2, and the hepatocyte-specific proteins CK18 and ALB were expressed at different stages of liver organoid differentiation. (D) The protein levels displayed in the histograms were determined by normalization to the levels of the internal control products GAPDH and β-actin. Data are shown as the mean±SEM (n=3). The scale bar is shown in the picture. DE, Definitive endoderm; FG, Foregut; iPSCs, human induced pluripotent stem cells; HLO: human liver organoid; ALB: albumin; CK18: cytokeratin 18; HNF4α, hepatocyte nuclear factor 4 alpha.

In this study, we established a differentiation protocol to transform hiPSCs into liver organoids by mimicking embryonic liver development. The protocol entailed the strategic addition of cytokines and small molecule compounds to the medium combined with Matrigel as a scaffold in 3D culture ( Figure 1A). Initially, the hiPSCs were treated with activin A and BMP4 for 3 days under 2D culture conditions to drive their differentiation into definitive endoderm (DE) cells [ 15, 16]. Subsequently, the DE cells were treated with CHIR99021 and FGF-10 for 3 days for differentiation into foregut endoderm (FG) cells [ 17, 18]. FG cells were then isolated into single cells and implanted into Matrigel and 3D cultured for 6‒7 days [ 17, 19]. During this period, spherical cell clusters composed of hepatic progenitor cells and irregular cell clusters composed of mesenchymal cells gradually appeared in the Matrigel. Finally, cells in Matrigel were cultured in hepatocyte medium (HCM) supplemented with dexamethasone, oncostatin M, and HGF, leading to the gradual formation of liver organoids containing multiple liver cell types and 3D tissue architecture as early as day 22 ( Figure 1A). Immunofluorescence (IF) and western blot (WB) analysis were used to detect the expressions of marker proteins at different stages of differentiation, further confirming the success of liver organoid differentiation ( Figure 1B‒D).

### Identification of the morphological structure and cellular components of liver organoids

**Figure 2:**
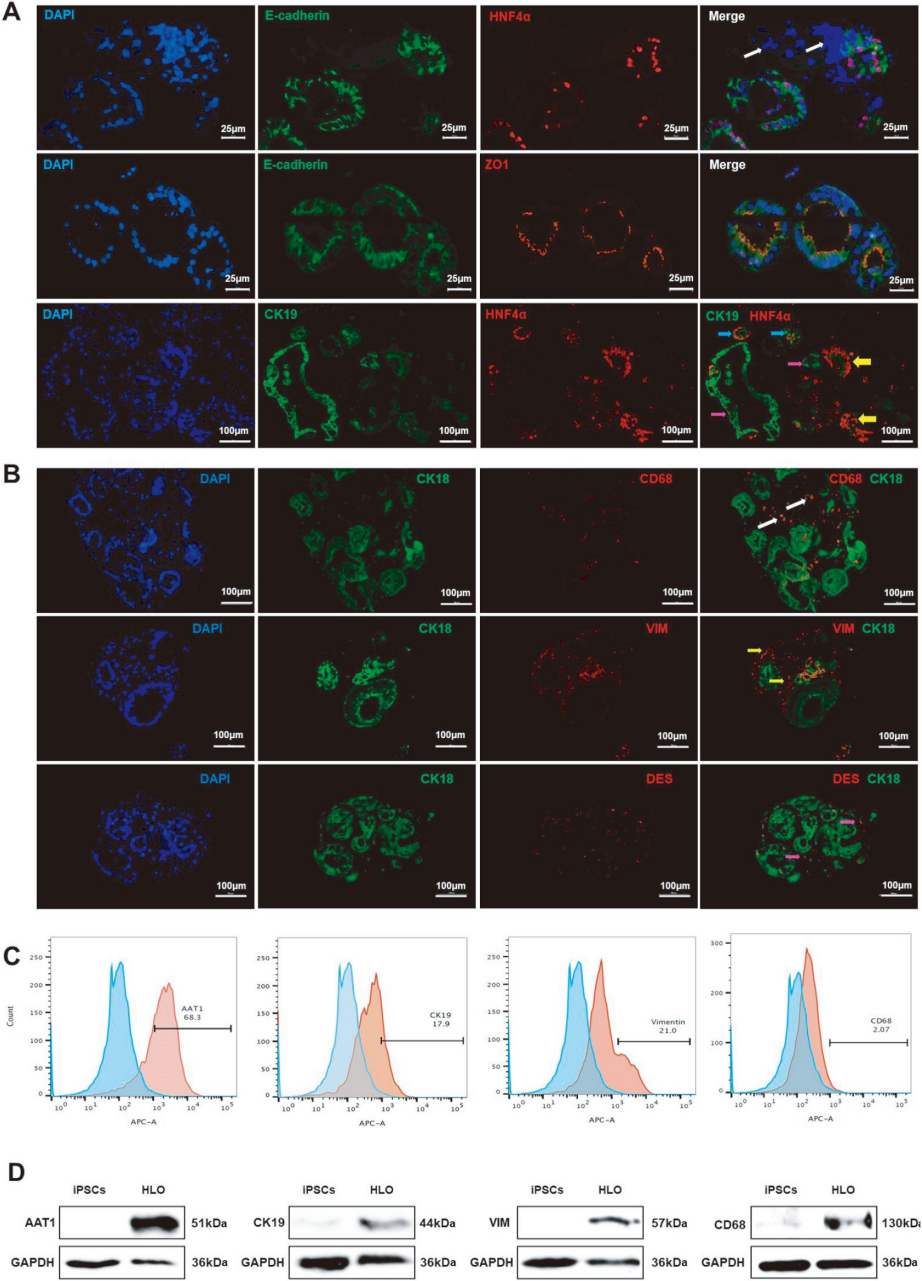
Figure 2 Immunofluorescence staining of liver organoid structure and cell composition (A) Immunofluorescence staining of paraffin sections showed that the spherical mass was composed of epithelial cells, and the irregular mass was composed of nonepithelial mesenchymal cells. White arrows indicate mesenchymal cells, yellow arrows indicate hepatocytes, purple arrows indicate cholangiocytes, and blue arrows indicate bipotent hepatic progenitor cells. (B) Paraffin section immunofluorescence staining showed that stromal cells expressed the Kupffer cell marker CD68 and hepatic stellate cell-specific proteins VIM and DES, as indicated by white arrows, yellow arrows, and purple arrows, respectively. (C) Flow cytometry confirmed the presence of CD68+, CK19+, AAT1+, and VIM+ cell populations in liver organoids. (D) Western blot analysis showed that the hepatocyte-specific protein AAT1, the cholangiocyte-specific protein CK19, the Kupffer cell marker protein CD68, and the hepatic stellate cell-specific protein VIM were expressed in liver organoids. The scale bar is shown in the picture. iPSCs, human induced pluripotent stem cells; HLOs, human liver organoids; AAT1, α1-antitrypsin; ZO-1, zonula occludens-1; VIM, vimentin; DES, desmin; CK18, cytokeratin 18; CK19, cytokeratin 19; HNF4α, hepatocyte nuclear factor 4 alpha.

Under the microscope, two types of structures were observed in the established liver organoids: spherical cell clusters and irregular cell clusters ( Figure 1A). However, in paraffin sections, irregular cell clusters are dispersed around spherical cell clusters due to their disrupted loose structure. Immunofluorescence staining was further conducted on these cell clusters to determine their structure and cellular components. First, paraffin sections of liver organoids were immunofluorescently stained for the hepatocyte marker hepatocyte nuclear factor (HNF4α), epithelial cell marker E-cadherin and cytoplasmic junction protein (zonula occludens-1, ZO-1). HNF4-α, a transcription factor that regulates a variety of liver genes, is located in the nucleus of hepatocytes and serves as a marker of hepatocyte lineages. E-cadherin, which is localized on the cell surface and at cell-to-cell junctions, is a marker of epithelial cells in the liver. ZO-1, a tight junction mucin, is mainly located in the lumen wall and is formed by epithelial cells. The staining results showed that the spherical cell clusters in the liver organoids were hollow saccular organoids composed of epithelial cells (hepatocytes and cholangiocytes) with polar epithelial structures ( Figure 2A). Furthermore, immunofluorescence staining with the cholangiocyte marker Cytokeratin 19 (CK19) demonstrated that some of the organoids with spherical cystic structures contained hepatocyte-like cells, some contained cholangiocyte-like cells, and some were composed of liver progenitor cells with bipotentials of hepatocytes and cholangiocytes ( Figure 2A).

Immunofluorescence staining revealed that the irregular cell clusters scattered around the spherical structure were negative for E-cadherin, ZO-1, and HNF4-α, indicating their nonepithelial mesenchymal nature. Further immunofluorescence staining revealed that these irregular stromal cell clusters expressed hepatic stellate cell-specific proteins or markers, including vimentin (VIM) and desmin (DES), as well as the Kupffer cell marker CD68 ( Figure 2B). Flow cytometry also detected the presence of liver-specific proteins, such as α1-antitrypsin (AAT1), CK19, VIM, and CD68, in the organoids ( Figure 2C). Western blot analysis further confirmed the expressions of HNF4-α, CK19, Vimentin, and CD68 in liver organoids ( Figure 2D). These findings indicated that, in addition to hepatic progenitor cells capable of differentiating into hepatocytes and cholangiocytes, the liver organoids we established also contained hepatic stellate cells and Kupffer cells.

### Assessment of liver-specific protein expression and characterization of liver-specific functions in liver organoids

**Figure 3:**
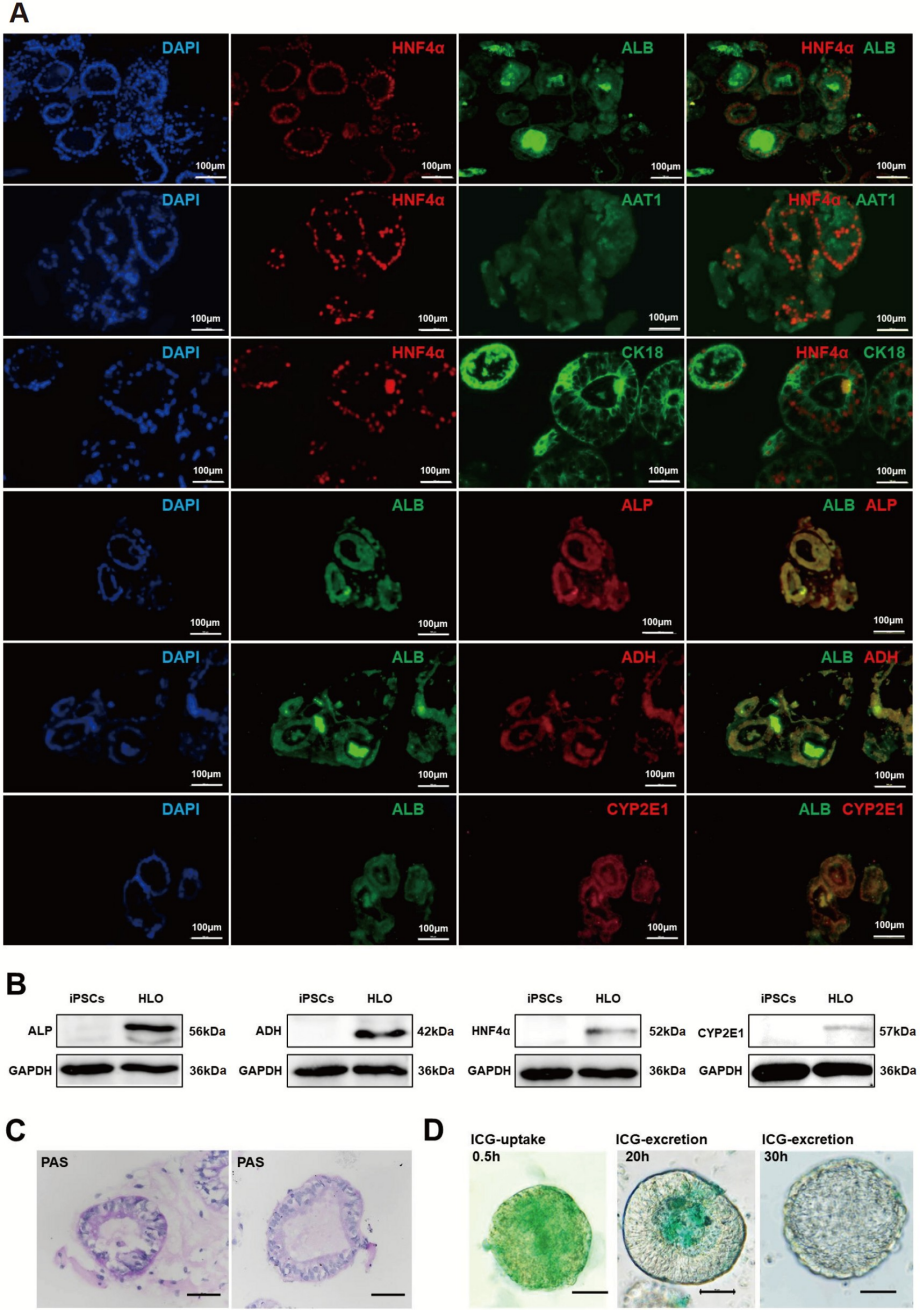
Figure 3 Detection of hepatocyte-specific proteins and liver-specific functions in liver organoids (A) Immunofluorescence staining of paraffin sections showing the expressions of the hepatocyte-specific proteins HNF4α, ALB, AAT1, CK18, ADH, ALP, and CYP2E1 in liver organoids. Scale bar: 100 μm. (B) Western blot analysis confirmed the expressions of the hepatocyte-specific proteins ALP, ADH, HNF4α, and CYP2E1 in liver organoids. (C) PAS staining of paraffin sections confirmed that liver organoids could synthesize and store glycogen. Scale bar: 50 μm. (D) ICG experiments confirmed that liver organoids can take up and excrete ICG. Scale bar: 50 μm. iPSCs, human induced pluripotent stem cells; HLO, human liver organoid; ALB, albumin; AAT1, α1-antitrypsin; CK18, cytokeratin 18; ADH, alcohol dehydrogenase; ALP, alkaline phosphatase; HNF4α, hepatocyte nuclear factor 4 alpha; CYP2E1, cytochrome P450 family member 2E1; PAS, periodic acid-Schiff; ICG, indocyanine green.

We subsequently performed immunofluorescence staining and western blot analysis to assess the expressions of liver-specific proteins in the liver organoids. The results demonstrated the presence of liver-specific proteins, including albumin (ALB), α1-antitrypsin (AAT1), cytokeratin 18 (CK18), alcohol dehydrogenase (ADH), alkaline phosphatase (ALP), and cytochrome P450 family member 2E1 (CYP2E1), in the liver organoids ( Figure 3A,B).

Subsequently, we performed periodic acid Schiff stain (PAS) and indocyanine green (ICG) uptake and release assays on liver organoids. PAS staining is mainly used to detect sugars in tissues and can indicate glycogen storage in liver organoid cells. PAS staining of liver organoid paraffin sections revealed positive staining in hepatocytes, indicating that liver organoids can synthesize glycogen ( Figure 3C). The ICG uptake and release assay assesses the functionality of hepatocytes by tracking the binding of ICG to albumin, its uptake by hepatocytes, and subsequent excretion. ICG experiments showed that ICG was rapidly taken up by hepatocytes in liver organoids within half an hour of treatment. Upon switching to normal HCM medium, ICG was gradually excreted from the cells and was completely removed after 30 h. This confirmed the presence of functional hepatocytes in the liver organoids with both ICG uptake and excretion capabilities ( Figure 3D).

### Establishment of an ALD model in liver organoids and analysis of associated gene expression

**Figure 4:**
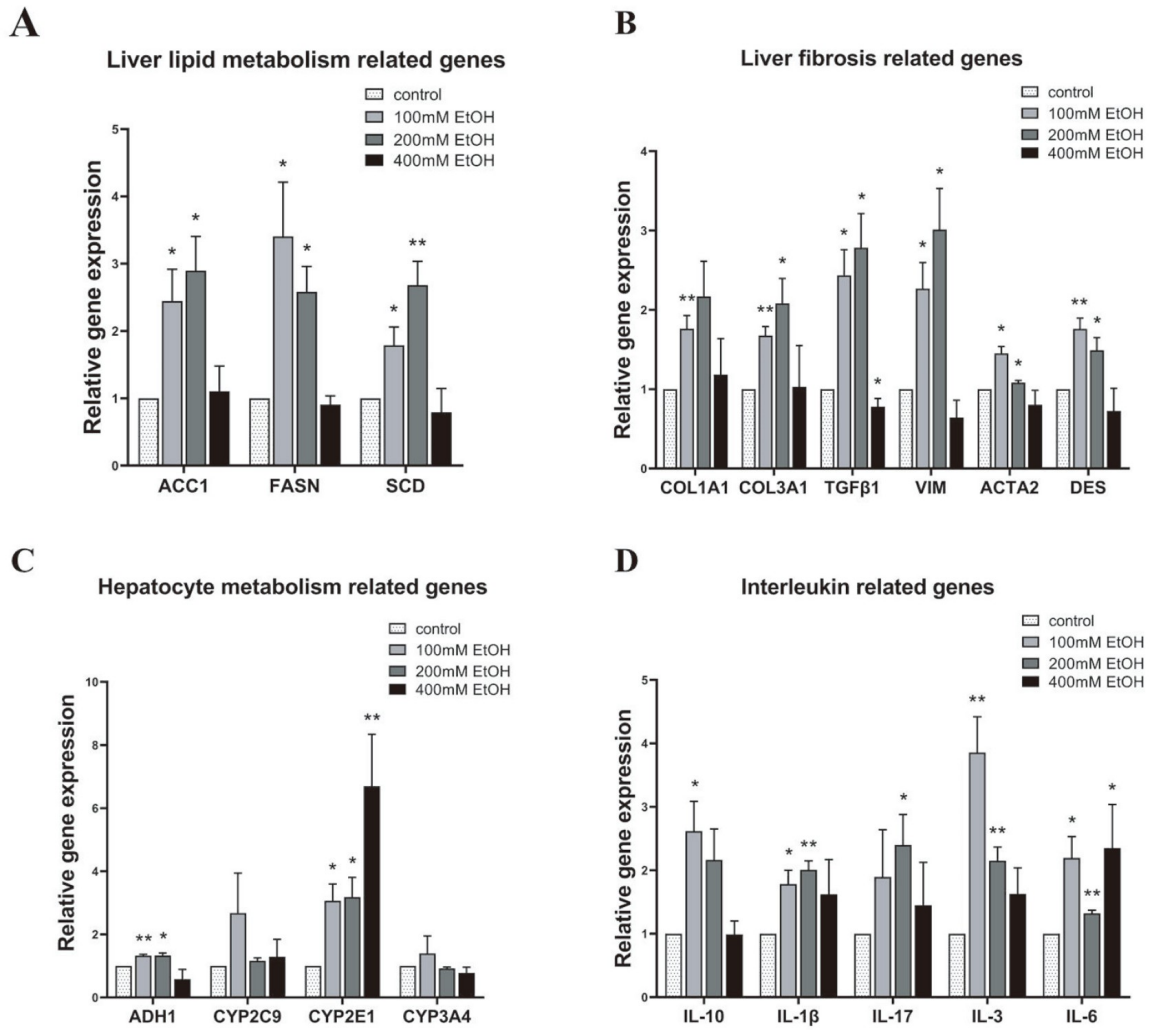
Figure 4 Gene expressions in liver organoids after treatment with different concentrations of ethanol The expression levels of liver lipid metabolism-related genes (A), liver fibrosis-related genes (B), hepatocyte metabolism-related genes (C), and interleukin-related genes (D) in liver organoids treated with different concentrations of ethanol were detected by qPCR and compared with the expression levels in liver organoids of the control group. GAPDH was used as an internal reference. Data are from 3 samples from 3 independent differentiation experiments and are shown as the mean±SEM. Analysis of variance was used to detect whether there was a significant difference in the gene expression levels among the groups. *P<0.05 and ** P<0.01. Control: liver organoids in the control group; EtOH: liver organoids in different ethanol concentration groups.

The ALD model was established by introducing different concentrations of ethanol into the culture medium between days 27 and 30 of liver organoid differentiation, while a control group (without ethanol) was maintained concurrently. Liver organoids were harvested after 72 h of ethanol treatment, and RNA was extracted to obtain the liver organoid cDNA library through reverse transcription PCR. The expressions of liver injury-related genes in the ALD model were assessed via fluorescence quantitative PCR and compared with that in the control group ( Figure 4). Treatment with 100 mM and 200 mM ethanol increased the expressions of the genes containing *ACC1*, *FASN*, and *SCD*, which are associated with lipid metabolism, in liver organoids. However, their expressions remained unchanged after treatment with 400 mM ethanol ( Figure 4A). Similarly, the expressions of the genes *COL1A1*, *COL3A1*, *ACTA2*, *DES*, *TGFβ1*, and *VIM*, which are involved in liver fibrosis, increased in liver organoids treated with 100 mM and 200 mM ethanol. Conversely, their expression levels remained unchanged and, in some cases, decreased following treatment with 400 mM ethanol ( Figure 4B). Furthermore, the expressions of the genes *ADH1* and *CYP2E1*, which encode enzymes involved in alcohol metabolism, increased in liver organoids after ethanol treatment. However, the expressions of genes of *CYP2C9* and *CYP3A4* remained unchanged ( Figure 4C). In addition, the expression levels of the interleukin-encoding genes *IL-10*, *IL-3*, *IL-6*, *IL-1β*, and *IL-17* in liver organoid cells increased to varying degrees following ethanol treatment ( Figure 4D). Nonetheless, due to potential variability between different experimental batches, some changes in gene expression did not reach statistical significance.

### Pathological changes in the ALD model

**Figure 5:**
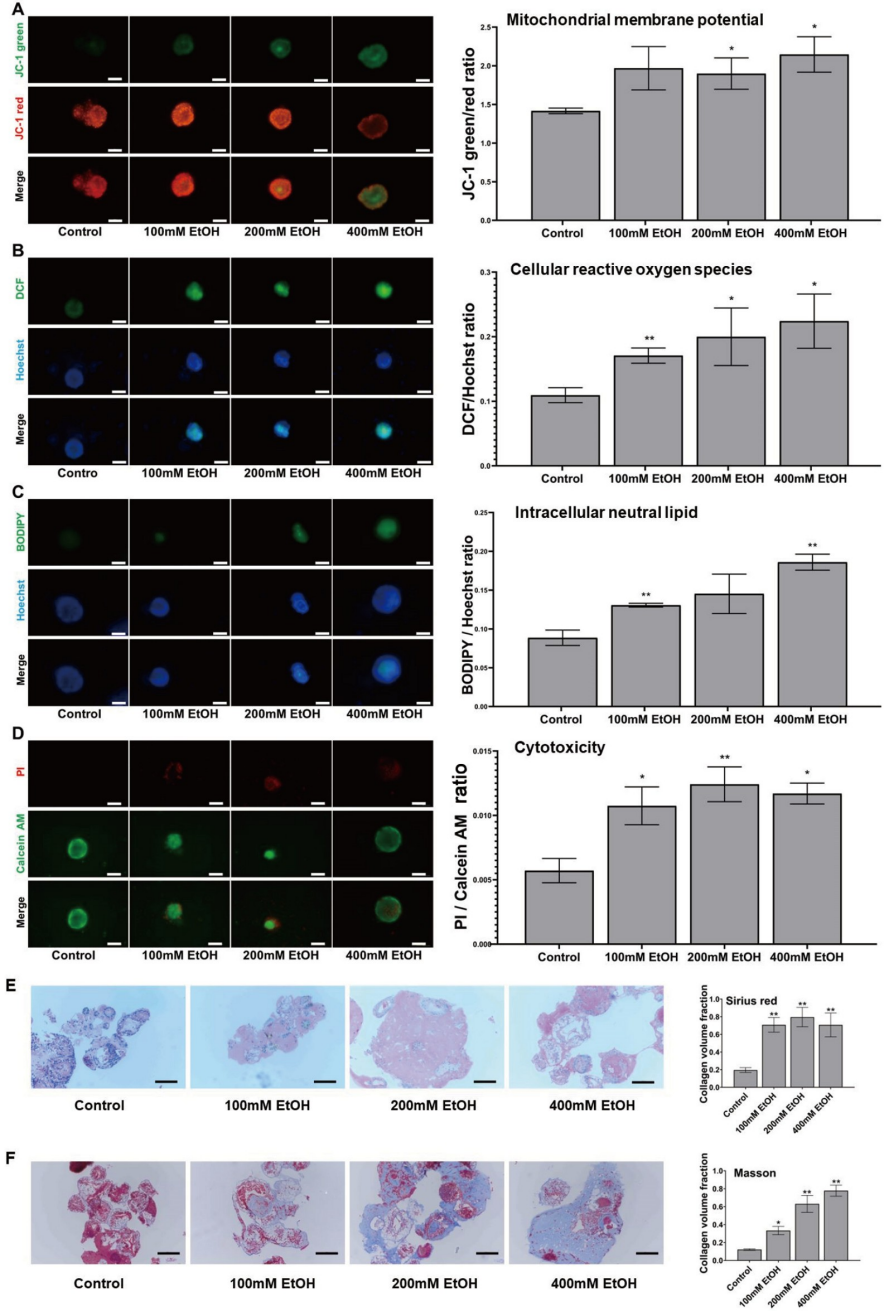
Figure 5 Pathological damage in the ALD model of liver organoids The MMP (A) and cellular ROS (B) levels in liver organoids after ethanol treatment for 24 h, neutral lipid levels (C) after ethanol treatment for 48 h, and the proportion of dead cells (D) after ethanol treatment for 72 h were detected and compared with the results for liver organoids in the control group. Left: images of HLOs under a fluorescence microscope. Scale bar: 100 μm. Right: graphs showing the fluorescence intensity ratios detected by a SpectraMax iD3 microplate reader. Data are presented as the mean±SEM (n=3 per group). Analysis of variance was used to detect whether there was a significant difference between each ethanol-treated group and the control group. *P<0.05 and **P<0.01. (E,F) The paraffin sections of liver organoids from the control and ethanol-treated groups were stained with Sirius red (E) and Masson’s trichrome (F). The collagen volume fractions are shown as the mean±SEM (n=3 per group). Analysis of variance was used to detect whether there was a significant difference between each ethanol-treated group and the control group. *P<0.05 and **P<0.01. Scale bar: 100 μm. Control: liver organoids in the control group; EtOH: liver organoids in different ethanol concentration groups.

The liver organoids were subjected to ethanol treatment to establish an ALD model. After 24 h of treatment, the mitochondrial membrane potential (MMP) and reactive oxygen species (ROS) levels in the model group were detected using a multifunction microplate reader and compared with those in the control group of liver organoids. After 24 h of ethanol treatment, the MMP decreased, and the ROS levels increased in the organoid cells ( Figure 5A,B). Consistently, under a fluorescence microscope, both JC green fluorescence intensity and DCF green fluorescence intensity in liver organoids increased after ethanol treatment, further confirming the decrease in the MMP and increase in ROS ( Figure 5A,B). After 48 h of ethanol treatment, the levels of neutral lipids in the model group were detected via BODIPY staining and compared with those in the control group of liver organoids. Fluorescence microscopy revealed an increase in BODIPY green fluorescence intensity in the liver organoids after ethanol treatment, indicating that the level of neutral lipids in the organoid cells increased ( Figure 5C). The results of the microplate reader also showed that after 48 h of ethanol treatment, the ratio of green/blue fluorescence intensity in liver organoids increased, indicating that the level of neutral lipids in organoid cells increased. After 72 h of ethanol treatment, the activity and death of cells in the organoids were detected by Calcein/PI cell viability and cytotoxicity detection kit. Under a fluorescence microscope, the red fluorescence intensity increased in the liver organoids after ethanol treatment, indicating that the number of dead cells increased in the organoids ( Figure 5D). Similarly, the results from the multimicroplate reader showed that the ratio of red/green fluorescence intensity in liver organoids increased after 72 h of ethanol treatment, indicating a decrease in organoid cell activity and an increase in the proportion of dead cells ( Figure 5D). Nonetheless, some changes may not have reached statistical significance, possibly due to significant errors between different experimental batches.

Furthermore, after 72 h of ethanol treatment, liver organoids from each group were harvested to prepare paraffin sections for Sirius red staining and Masson staining. The results showed an increased collagen volume fraction in liver organoids following ethanol treatment ( Figure 5E,F).

### The therapeutic impact of drugs on the liver organoid model of ALD

Finally, we assessed this model with three drugs reported in the literature to have therapeutic effects on ALD to verify its suitability for drug screening. On the 27th to 30th days of liver organoid differentiation, 100 mM ethanol was added to the culture medium to establish the ALD model. Moreover, three low, medium, and high doses of IL-22, metadoxine (Met) and N-acetylcysteine (NAC) were added to the medium to treat the ALD model. After three days, the therapeutic effect of the drug on the ALD model was assessed using Calcein/PI cell viability and cytotoxicity detection kit and a multifunctional microplate reader. The toxicity of the drug to liver organoids was also evaluated.

**Figure 6:**
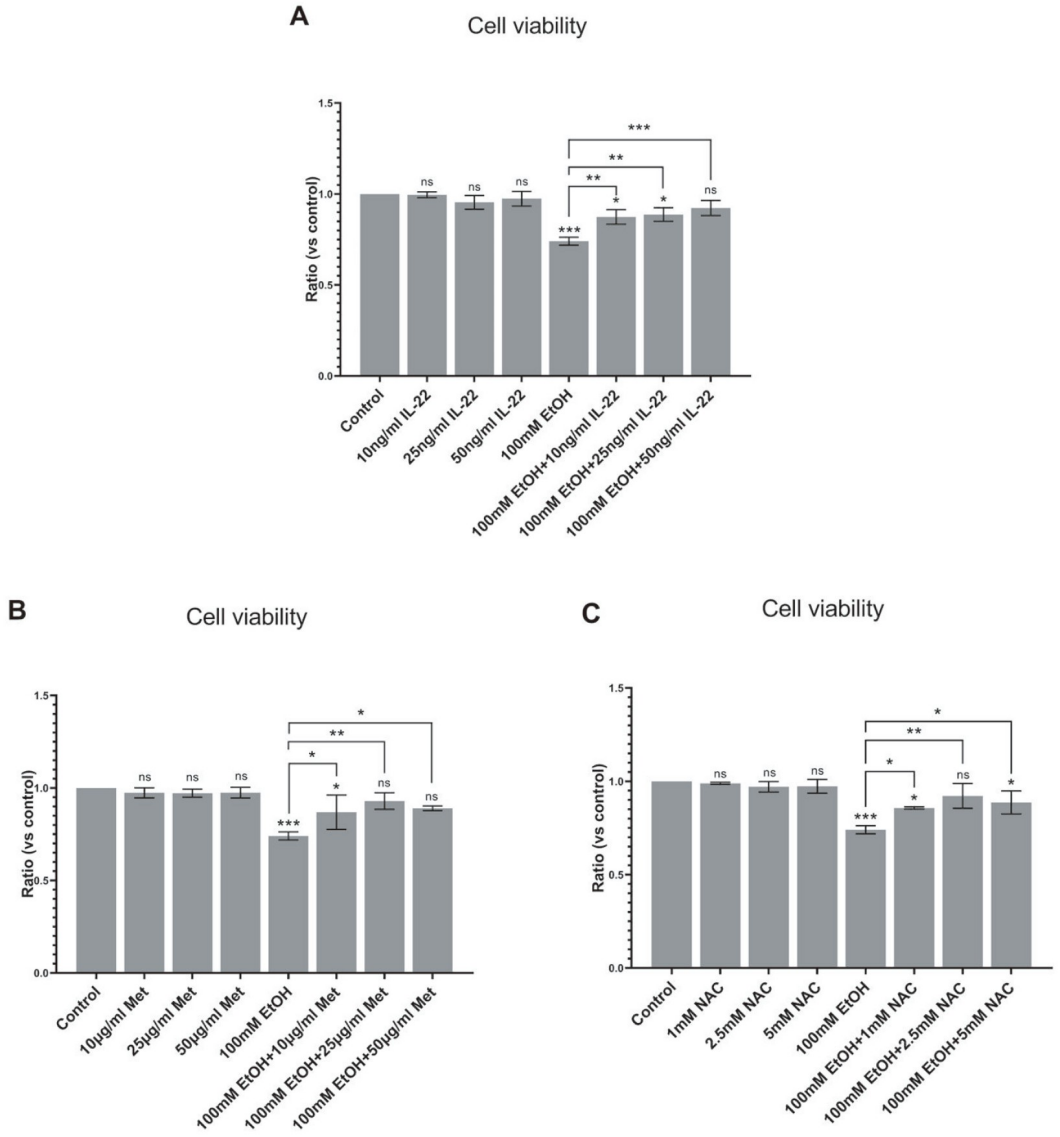
Figure 6 Drug treatment of ALD models The ALD liver organoid model was established with 100 mM ethanol, and the model was treated with different doses of IL-22, Met, or NAC. Three days later, the therapeutic effect was detected with a calcein/PI cell viability and cytotoxicity detection kit, and the toxicity of the three drugs to liver organoids was also detected. (A) The ALD model was treated with 10, 25, or 50 ng/mL IL-22. (B) The ALD model was treated with Met at 10, 25, or 50 μg/mL. (C) The ALD model was treated with NAC at 1, 2.5, or 5 mM. Data are from 3 independent samples and are shown as the mean±SEM. Analysis of variance was used to detect whether there was a significant difference between each treatment group and the control group or untreated ALD group. ns: not statistically significant. *P<0.05, **P<0.01, and ***P<0.001. IL-22: interleukin 22; Met: metadoxine; NAC: N-acetylcysteine; Control: liver organoids in the control group; EtOH: liver organoids in different ethanol concentration groups.

The results indicated that the cell viability of liver organoids after treatment with the three drugs did not significantly differ from that of the control group of liver organoids without drug treatment, suggesting that these drugs at their respective doses had no toxic effects on liver organoids. After treatment with 100 mM ethanol, the proportion of dead cells in the liver organoids increased, and the cell viability decreased significantly, confirming the successful establishment of the ALD model ( Figure 6). The cell viability of the IL-22 treatment group was greater than that of the untreated group, and the cell viability of the high-dose group was not different from that of the control group, indicating that IL-22 exerted preventive and therapeutic effects on ALD, with a more pronounced effect at high doses ( Figure 6A). Similarly, the cell viability of the Met treatment group was also greater than that of the untreated group. Compared with those in the control group, the cell viability in the middle- and high-dose groups differed, indicating that Met had preventive and therapeutic effects on ALD, with the medium-dose treatment showing the most significant therapeutic effect ( Figure 6B). The cell activity in the NAC treatment group was greater than that in the untreated group, with the medium-dose group showing no difference from the control group. This finding suggested that NAC had both preventive and therapeutic effects on ALD, with the medium dose being the most effective ( Figure 6C).

## Discussion

The liver, the largest solid organ in the human body, plays a crucial role in metabolism [20]. Liver diseases have emerged as a prominent cause of mortality worldwide [21]. Among these diseases, alcoholic liver disease (ALD) is a common liver disease [1]. Nevertheless, advancements in ALD research are impeded by the lack of suitable disease models. In laboratories, researchers often establish ALD models in rats or mice through intragastric alcohol administration, alcohol consumption, or intraperitoneal alcohol injection [ 5, 6]. Although these models can replicate some pathological manifestations of human alcoholic liver injury, such as hepatocyte steatosis, liver necrosis, apoptosis and inflammation, species-specific variations persist [ 5, 6]. The metabolic pathways of alcohol in humans and model animals are not entirely the same, as is their response to liver injury [8]. Therefore, findings from animal models may not fully reflect the complexities of human physiology, limiting their use in ALD research [8].

In addition, human liver cell lines, human liver cancer cell lines, and primary human hepatocytes (PHHs) have also been used to establish ALD models. Typically, these models involve the addition of ethanol to the cell culture medium to mimic the impact of alcohol on liver cells *in vivo* [ 22– 24]. However, these 2D cultured cells exhibit limitations. They consist of a single component, lack intricate cell interactions, and fail to replicate the native characteristics of the liver microenvironment. Furthermore, once PHHs are removed from their *in vivo* microenvironment, they rapidly degenerate, lose their original functionality, and experience diminished proliferative capacity [25]. Liver cell lines are difficult to culture, and recent reports have raised concerns about contamination with HeLa cell lines [26], questioning the reliability of the experimental results. Compared with normal liver cells, liver cancer cell lines are abnormal cells with functional disparities, necessitating cautious interpretation of the results. Consequently, these 2D cultured cell models face challenges in reproducing the pathological manifestations of ALD *in vivo*. These limitations, combined with individual differences in susceptibility to human ALD, limit the use of these cell models [ 9, 10].

Moreover, the intricate interplay among hepatocytes, immune cells, and stellate cells, which are critical in the pathogenesis of ALD [ 1, 27], presents a challenge for *in vitro* culture models composed of these individual cell components. Such models struggle to faithfully replicate the full spectrum of pathological changes observed in alcoholic liver injury *in vivo*. While some researchers have cocultured hepatocytes with liver nonparenchymal cells to simulate liver function and the microenvironment [ 28, 29], this approach introduces complexity to cell culture. In a previous study, researchers co-cultured human liver organoids differentiated from human embryonic stem cells (ESCs) with human fetal liver mesenchymal cells (hFLMCs) in 3D culture and established an ALD model by ethanol treatment [30]. This ALD model contains a variety of liver cell components and effectively recapitulates certain pathological manifestations of ALD [30]. However, it is important to note that the study is technically complicated and time-consuming, and hFLMCs are not easy to obtain, significantly limiting their application.

In this study, we employed a sophisticated approach involving the introduction of cytokines and small molecule compounds to the cell culture medium combined with 3D culture. This method allowed us to successfully differentiate hiPSCs into liver organoids in approximately 22 days. These liver organoids exhibited a comprehensive composition, containing hepatocyte-like cells, cholangiocyte-like cells, hepatic stellate cells, and Kupffer cells. Various studies have confirmed that liver organoids possess liver cell polarity and multiple liver-specific functions and express various liver-related proteins. Subsequently, we harnessed this advanced liver organoid platform to establish a human ALD model by treating liver organoids with ethanol. By fluorescence quantitative PCR, we found that genes related to ALD-related liver injury, such as alcohol metabolism genes, lipid metabolism genes, fibrosis-related genes, and inflammation-related interleukin genes, were upregulated in the ALD liver organoid model. In hepatocytes, ethanol is mainly metabolized by alcohol dehydrogenase and CYP2E1 to acetaldehyde, which is responsible for the generation of ROS. ROS cause oxidative stress and steatosis, which lead to inflammation and apoptosis [ 1, 4, 31]. Ethanol can also induce liver cell apoptosis by activating the intrinsic mitochondrial apoptosis pathway. Furthermore, hepatocyte death, followed by the release of damage-associated molecular patterns (DAMPs), can activate Kupffer cells [1]. Activated Kupffer cells produce many proinflammatory cytokines (for example, interleukins) that promote HSC activation. ROS and acetaldehyde can also directly stimulate HSCs, resulting in fibrogenesis [4]. Remarkably, our model recapitulated key pathological features of ALD *in vitro*, including hepatocyte steatosis, mitochondrial damage, increased ROS levels, hepatocyte necrosis, and fibrosis. Finally, we assessed this model with three drugs reported in the literature to have therapeutic effects on ALD. NAC and metadoxine are antioxidants that can counteract the oxidative effects caused by ethanol [ 32, 33]. IL-22 can induce the expressions of proliferation genes and reduce the expressions of apoptosis genes in hepatocytes [34]. The screening results revealed the therapeutic effects of these three drugs on ALD, confirming that the model is suitable for large-scale drug screening.

Evidence suggests that susceptibility to ALD is influenced by genetic factors and that pathological processes and drug responses are individual-specific [35]. To address this variability, liver organoids derived from patient-specific hiPSCs are particularly valuable. These organoids carry the patient’s genetic information, providing a more accurate reflection of how the patient’s liver responds to alcohol and drugs. Additionally, since they are not rejected by the body’s immune system, hiPSC-derived liver organoids can be used as a potential source of cell therapy and for liver transplantation. Finally, liver organoids are suitable for large-scale engineering culture [ 11, 13]. Therefore, liver organoids established from patient-derived hiPSCs are more suitable as a drug screening platform for ALD and can be used to evaluate the efficacy and toxicity of candidate drugs and predict the possibility of severe side effects. Taken together, these advantages underscore the immense potential of hiPSC-derived liver organoids in personalized drug development and liver transplantation therapy.

## Supporting information

24050Supplementary_Tables

## Supplementary Data

Supplementary data is available at *Acta Biochimica et Biophysica Sinica* online.
