# Supplementary material for: Development of an alcoholic liver disease model for drug evaluation from human induced pluripotent stem cell-derived liver organoids: ALD model from hiPSC-derived liver organoids
Source: Acta Biochim Biophys Sin (Shanghai). 2024 May 30;56(10):1460–72. doi: 10.3724/abbs.2024074 (PMC11532202; doi:10.3724/abbs.2024074)
Supplement: 24050Supplementary_Tables [file 24050Supplementary_Tables.pdf]

Supplementary Table S1. The sequences of primers used in the quantitative polymerase chain reaction

Gene	Forward primer sequence (5'⟶3')	Reverse primer sequence (5'⟶3')
h-VIM	GCCCTAGACGAACTGGGTC	GGCTGCAACTGCCTAATGAG
h-CYP2C9	CAGAGACGACAAGCACAACCCT	ATGTGGCTCCTGTCTTGCATGC
h-CYP2E1	ATGTCTGCCCTCGGAGTCA	CGATGATGGGAAGCGGGAAA
h-CYP3A4	CACGAGCAGTGTTCTCTCCTT	CACAGTATCATAGGTGGGTGGT
h-ADH1	AGTCATCCCACTCGCTATTCC	GTCCCCTGAGGATTGCTTACA
h-COL1A1	GAGGGCCAAGACGAAGACATC	CAGATCACGTCATCGCACAAC
h-COL3A1	GCCAAATATGTGTCTGTGACTCA	GGGCGAGTAGGAGCAGTTG
h-LOXL2	GGGTGGAGGTGTACTATGATGG	CTTGCCGTAGGAGGAGCTG
h-TGF β1	CTAATGGTGGAAACCCACAACG	TATCGCCAGGAATTGTTGCTG
h-ACC1	TACAGGCTGGCTCAGGACTAT	CGCAACATTTTGTAGCACTCTG
h-FASN	ACAGCGGGGAATGGGTACT	GACTGGTACAACGAGCGGAT
h-SCD	TTCCTACCTGCAAGTTCTACACC	CCGAGCTTTGTAAGAGCGGT
h-IL-3	CAGACAACGCCCTTGAAGACA	GCCCTGTTGAATGCCTCCA
h-IL-1β	ATGATGGCTTATTACAGTGGCAA	GTCGGAGATTCGTAGCTGGA
h-IL-17	AGATTACTACAACCGATCCACCT	GGGGACAGAGTTCATGTGGTA
h-IL-10	TCAAGGCGCATGTGAACTCC	GATGTCAAACTCACTCATGGCT
h-IL-6	ACTCACCTCTTCAGAACGAATTG	CCATCTTTGGAAGGTTCAGGTTG

Supplementary Table S2. Antibodies used in immunofluorescence staining (IF), flow cytometry (F) and western blot analysis (WB)

Antibody	Company (Cat.)	Application (dilution)
ZO-1 (D6L1E) Rabbit mAb	CST (#13663)	IF (1:400)
Nanog (D73G4) XP® Rabbit mAb	CST (#4903)	IF (1:200), WB (1:2000)
Oct-4 (D7O5Z) Mouse mAb	CST (#75463)	IF (1:200), WB (1:1000)
Sox17 (D1T8M) Rabbit mAb	CST (#81778)	IF (1:3200), WB (1:1000)
CDX2 (D11D10) Rabbit mAb	CST (#12306)	IF (1:200), WB (1:1000)
CD68 (D4B9C) XP® Rabbit mAb	CST (# 76437)	IF (1:800), WB (1:1000), F (1:200)
Vimentin (D21H3) XP® Rabbit mAb	CST (# 5741)	IF (1:200), WB (1:1000), F (1:50)
Desmin (D93F5) XP® Rabbit mAb	CST (# 5332)	IF (1:100)
HNF4α (C11F12) Rabbit mAb	CST (#3113)	IF (1:6000), WB (1:1000)
Rabbit Anti-CYP2E1 antibody	Bioss (bs-4562R)	IF (1:100), WB (1:1000)
Mouse Anti-Cytokeratin 19 antibody	Bioss (bsm-33057M)	IF (1:200), WB (1:1000), F (1:100)
Mouse Anti-Cytokeratin 18 antibody	Bioss (bsm-33103M)	IF (1:500), WB (1:1000)
Alpha 1 Antitrypsin Monoclonal antibody (Mouse / IgG1)	Proteintech (66135-1-Ig)	IF (1:100), WB (1:1000), F (1:100)
Albumin Polyclonal antibody (Rabbit / IgG)	Proteintech (66051-1-Ig)	IF (1:500), WB (1:2000)
Anti-Alcohol Dehydrogenase antibody (Rabbit monoclonal)	Abcam (ab108203)	IF (1:500), WB (1:1000)
Rabbit Anti-ALP antibody	Bioss (bs-1535R)	IF (1:200), WB (1:1000)
DyLight 550 Conjugated AffiniPure Goat Anti-mouse IgG	BOSTER (BA1133)	IF (1:200)
DyLight 550 Conjugated AffiniPure Goat Anti-rabbit IgG	BOSTER (BA1135)	IF (1:200)
FITC Conjugated AffiniPure Goat Anti-rabbit IgG	BOSTER (BA1105)	IF (1:200)
DyLight 488 Conjugated AffiniPure Goat Anti-mouse IgG	BOSTER (BA1126)	IF (1:200)
Goat anti-Rabbit IgG (H+L) Cross-Adsorbed Secondary Antibody, Alexa Fluor 647	Thermo (A-21244)	F (1:500)
Goat anti-Mouse IgG (H+L) Cross-Adsorbed Secondary Antibody, Alexa Fluor 647	Thermo (A-21235)	F (1:500)
Recombinant Anti- GAPDH antibody (Rabbit mAb)	Servicebio (GB15004)	WB (1:1000)
Goat Anti-Rabbit Mouse IgG-HRP	Abmart (M21003)	WB (1:7000)
Antifade Mounting Medium	Absin (abs9234)	IF
DAPI mounting medium	Abcam (ab104139)	IF
